# Findings from the SASA! Study: a cluster randomized controlled trial to assess the impact of a community mobilization intervention to prevent violence against women and reduce HIV risk in Kampala, Uganda

**DOI:** 10.1186/s12916-014-0122-5

**Published:** 2014-07-31

**Authors:** Tanya Abramsky, Karen Devries, Ligia Kiss, Janet Nakuti, Nambusi Kyegombe, Elizabeth Starmann, Bonnie Cundill, Leilani Francisco, Dan Kaye, Tina Musuya, Lori Michau, Charlotte Watts

**Affiliations:** Gender Violence and Health Centre, London School of Hygiene and Tropical Medicine, 15-17 Tavistock Place, London, WC1H 9SH UK; Raising Voices, 16 Tufnell Drive, Kamwokya, P.O. Box 6770, Kampala, Uganda; Department of International Health, Johns Hopkins Bloomberg School of Public Health, 615 N. Wolfe Street, Baltimore, MD 21205 USA; Department of Obstetrics and Gynaecology, School of Medicine, Makerere University, P.O. Box 7072, Kampala, Uganda; Centre for Domestic Violence Prevention, 16 Tufnell Drive, Kamwokya, P.O. Box 6770, Kampala, Uganda

**Keywords:** Violence prevention, Impact evaluation, Community mobilization, Intimate partner violence, Uganda, HIV, Gender based violence, East Africa

## Abstract

**Background:**

Intimate partner violence (IPV) and HIV are important and interconnected public health concerns. While it is recognized that they share common social drivers, there is limited evidence surrounding the potential of community interventions to reduce violence and HIV risk at the community level. The SASA! study assessed the community-level impact of SASA!, a community mobilization intervention to prevent violence and reduce HIV-risk behaviors.

**Methods:**

From 2007 to 2012 a pair-matched cluster randomized controlled trial (CRT) was conducted in eight communities (four intervention and four control) in Kampala, Uganda. Cross-sectional surveys of a random sample of community members, 18- to 49-years old, were undertaken at baseline (n = 1,583) and four years post intervention implementation (n = 2,532). Six violence and HIV-related primary outcomes were defined *a priori*. An adjusted cluster-level intention-to-treat analysis compared outcomes in intervention and control communities at follow-up.

**Results:**

The intervention was associated with significantly lower social acceptance of IPV among women (adjusted risk ratio 0.54, 95% confidence interval (CI) 0.38 to 0.79) and lower acceptance among men (0.13, 95% CI 0.01 to 1.15); significantly greater acceptance that a woman can refuse sex among women (1.28, 95% CI 1.07 to 1.52) and men (1.31, 95% CI 1.00 to 1.70); 52% lower past year experience of physical IPV among women (0.48, 95% CI 0.16 to 1.39); and lower levels of past year experience of sexual IPV (0.76, 95% CI 0.33 to 1.72). Women experiencing violence in intervention communities were more likely to receive supportive community responses. Reported past year sexual concurrency by men was significantly lower in intervention compared to control communities (0.57, 95% CI 0.36 to 0.91).

**Conclusions:**

This is the first CRT in sub-Saharan Africa to assess the community impact of a mobilization program on the social acceptability of IPV, the past year prevalence of IPV and levels of sexual concurrency. SASA! achieved important community impacts, and is now being delivered in control communities and replicated in 15 countries.

**Trial registration:**

ClinicalTrials.gov #NCT00790959,

Study protocol available at http://www.trialsjournal.com/content/13/1/96

**Electronic supplementary material:**

The online version of this article (doi:10.1186/s12916-014-0122-5) contains supplementary material, which is available to authorized users.

## Background

### Background and study rationale

Violence against women is recognized as an important public health, social policy and human rights concern. Recent global estimates suggest that 30% of women will experience physical or sexual violence from an intimate partner during their lifetime [1], with far reaching consequences for their physical, mental and emotional health [2,3]. Several recent studies have also identified intimate partner violence (IPV) as an independent risk factor for HIV infection [4–6].

Underlying both women’s risk of IPV and HIV, and the associations between them, is gender inequality – women’s lower socioeconomic and political status, unequal access to education and employment, and a range of gender norms that both perpetuate and result from this inequality [7]. There is increasing evidence that the high levels of IPV documented in many settings are in part due to gender norms that support men’s dominance and control of women, create expectations about sexual entitlement for men and promote women’s subservience and obedience to men. These norms and power inequalities often limit the extent to which women can negotiate the circumstances of sex or insist on condom use, especially where violence or the threat of violence is commonplace. This, in turn, reduces their ability to protect themselves from HIV infection [4,8]. The gendered nature of the HIV/AIDS epidemic is particularly apparent in sub-Saharan Africa, where women and girls now constitute 58% of those living with the virus [7]. Furthermore, gender and power inequalities may increase women’s risk of violence following a diagnosis of HIV, which may in turn reduce women’s willingness and ability to test for HIV, disclose their status or seek treatment [7,9,10].

The need for HIV prevention efforts to more explicitly incorporate program elements to address gender inequality and violence has been repeatedly articulated, and the elimination of sexual and gender-based violence has been identified by the Joint United Nations Program on HIV/AIDS (UNAIDS) as being one of the core pillars of HIV prevention [11]. Despite this rhetoric however, the prevention of HIV and IPV often remain separate, and there has been relatively limited investment in prevention strategies that seek to tackle their shared, more upstream structural determinants.

The field of violence prevention research is in its relative infancy. A small number of rigorous trials have sought to evaluate the impact of violence and HIV prevention interventions in sub-Saharan Africa, with some promising results. Reductions in past year IPV and indicators of HIV-risk have been demonstrated in relation to a combined microfinance and gender/HIV training intervention in rural South Africa [12,13], a participatory HIV-prevention program in the Eastern Cape Province of South Africa [14] and gender dialogue groups added to a group savings program in rural Cote d’Ivoire [15]. However, each of these interventions has been primarily targeted towards enrolled individuals, and their evaluations have thus focused on individual-level impact. As a consequence they provide limited insights into how broader community level change can be achieved. To help address this gap, this paper presents the findings on the primary outcomes of the SASA! study, a cluster randomized controlled trial to assess the community-level impacts of SASA!, a community mobilization intervention seeking to prevent violence against women and reduce HIV-risk behaviors in Kampala, Uganda.

## Methods

### Study population

The study was conducted between November 2007 and May 2012 in the Rubaga and Makindye Divisions of Kampala, Uganda. Kampala has a high prevalence of IPV and HIV/AIDS. Of women 15- to 49-years old, 9.5% are estimated to be living with HIV [16] and, while this represents a marked decline since the epidemic peaked in Uganda in the early 1990s (reaching a prevalence of 21.1% among pregnant women attending antenatal clinics in 1991), studies suggest that incidence may again be on the rise [17,18]. Furthermore, in the 2011 Demographic and Health Survey (DHS) data from Kampala, 45% of ever-married women, 15- to 49-years old, reported lifetime experience of physical and/or sexual violence by their current or most recent partner [19].

### The SASA! intervention

The *SASA! Activist Kit for Preventing Violence against Women and HIV* [20] is a community mobilization intervention that seeks to change community attitudes, norms and behaviors that result in gender inequality, violence and increased HIV vulnerability for women. SASA! was designed by Raising Voices and was implemented in Kampala by the Centre for Domestic Violence Prevention (CEDOVIP). Designed around the Ecological Model of violence [21,22] SASA! recognizes that IPV results from the complex interplay of factors which operate at the individual, relationship, community and societal levels, and, therefore, systematically involves a broad range of stakeholders within the community including community activists, local governmental and cultural leaders, professionals such as police officers and health care providers, and institutional leaders. The central focus of the intervention is to promote a critical analysis and discussion of power and power inequalities - not only of the ways in which men and women may misuse power and the consequences of this for their intimate relationships and communities, but also on how people can use their power positively to affect and sustain change at an individual and community level.

**Figure 1:**
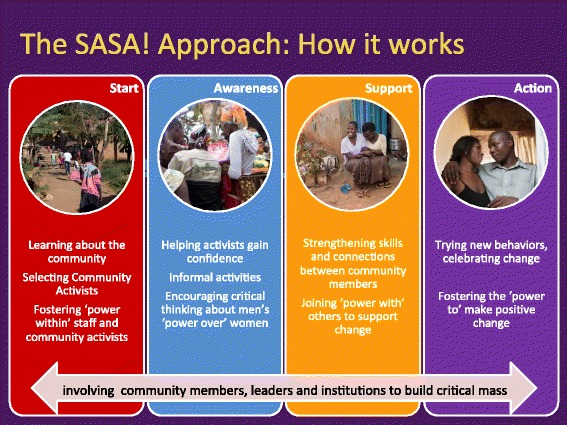
**Four phases of SASA!**

SASA!, which means ‘now’ in Kiswahili, is also an acronym for the phases of the approach: Start, Awareness, Support, Action which structure and systematize the community mobilization efforts (see Figure 1). In Start, community activists (CAs) (regular women and men) interested in issues of violence, power and rights are selected and trained, along with staff from selected institutions (for example, police, health care, and so on). Initially, eight CAs per parish were recruited (forming the basis of our sampling frame as discussed below), though no limits were set on how many others became involved during the natural course of intervention implementation. This cadre of activists then work through the Awareness, Support and Action phases of SASA!, introducing new concepts of power and encouraging an analysis of the imbalance of power through four strategies: Local Activism, Media and Advocacy, Communication Materials, and Training. The CAs conduct informal activities within their own social networks, fostering involvement and activism among their families, friends, colleagues and neighbors. The specifics of intervention activities are not rigidly proscribed but rather develop and evolve in direct response to community priorities, needs and characteristics. Each phase builds on the other, with an increasing number of individuals and groups involved, strengthening a critical mass committed to and able to create social norm change. Owing to the requirements of the trial design, the media and advocacy activities were restricted to local media channels in order to try to avoid exposing control communities to SASA! ideas and materials [see Additional file 1].

### Intervention logic model

**Figure 2:**
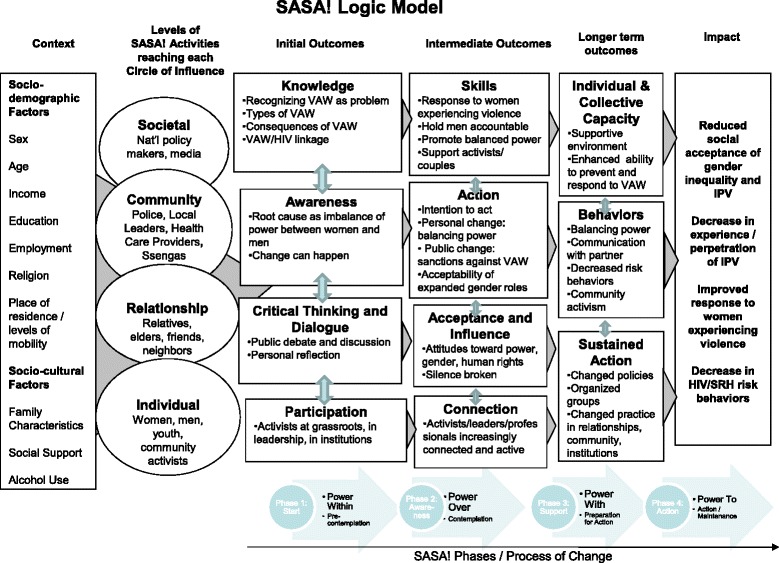
**SASA logic model.**

The intervention logic model (Figure 2) maps out the key contextual variables that may influence intervention impact; the levels of SASA! activities conducted in different spheres of influence; the expected initial, intermediate and longer term outcomes of the intervention; and the long-term sustained impact the intervention is designed to have on the community [see Additional file 2 for further details].

### Evaluation design

The study employed a cluster-randomized design, with randomization carried out within matched pairs. Full details of study design are presented in the SASA! Study protocol [23]. Briefly, eight ‘sites’ eligible for delivery of the intervention (each comprising one or two administrative Parishes) were identified on the basis of operational and programmatic considerations. All sites were separated from each other by a geographical buffer (at least one parish wide) to reduce the potential for intervention diffusion into control sites. Sites were matched into four pairs on the basis of qualitative assessments by CEDOVIP staff as to whether the site was urban or peri-urban, and the stability/mobility of the local population. Randomization was carried out by the research team in September 2007. The names of the two communities within a matched pair were written on identical pieces of paper which were then folded and put in a bag. One paper was blindly drawn from the bag, the selected name was assigned as an intervention community, and the other designated as a control. Control sites were waitlisted to receive the full intervention upon study completion. However, because of how pre-existing services are organized, police and healthcare provider engagement took place across intervention and control sites. The SASA! study thus examines the added value of the intensive local components of the intervention when implemented against this backdrop of involvement with these sectors, rather than the impact of the whole package versus nothing.

A baseline cross-sectional survey of community members was conducted in intervention and control communities prior to intervention implementation to provide information on the study communities, and to assess the underlying comparability of intervention and control communities. A follow-up cross-sectional survey using the same methodology took place four years later (January to May 2012). Barriers to program activity during the follow-up period (due to political disturbances and the suspension of activities during political election campaigns) mean that this four year follow-up equates to approximately 2.8 years of SASA! programming (discussed further below).

The sampling frame for the two cross-sectional surveys was drawn up to represent the population most likely to have had repeated and extensive contact with intervention activities. Multistage stratified random sampling (described elsewhere) was used to sample community members living in close proximity to (the same Enumeration Areas (EAs) as) CAs [23]. In control sites, ‘passive’ volunteers, recruited using an identical process as that used to recruit CAs in intervention sites, were used as the foci for sampling. The same sampling frame (though with updated household lists) was used at follow-up, with no sampling substitutions made where CAs had moved away, been substituted or been lost for other reasons. For reasons of safety and logistics, the sample was exclusively female around female activists and male around male activists. A person was eligible for inclusion in the survey if they usually lived in the household and shared food, had lived in the area for at least a year, and were 18-to 49-years old. A limit of one respondent per household was set out of consideration for respondent safety and confidentiality.

The study was conducted in accordance with WHO guidelines for the safe and ethical collection of data on violence against women [24]. These guidelines seek to minimize reporting biases and risk of harm to both respondents and interviewers. At both baseline and follow-up, interviewers received at least three weeks of training on the ethical and methodological issues surrounding the conduct of a survey relating to IPV and HIV, as well as ongoing support during the course of the survey. Interviewers were all from the local area, and interviewed respondents of the same sex as themselves. Interviews were conducted in private settings, in Luganda or English, and were concluded by providing information on additional support services in the area. At baseline, interviewers conducting the baseline survey were blinded as to the allocation of the intervention. It was not, however, possible to keep follow-up interviewers blinded.

The study received ethical approval from institutional review boards at the London School of Hygiene and Tropical Medicine (UK) (ref.5210), Makerere University (Uganda) (ref. 2007-101) and the Uganda National Council for Science and Technology (SS 2048). Approval to work in the study communities was obtained from local government offices and leaders, while individual-level written consent was obtained prior to each interview.

### Outcomes

Primary outcomes were selected *a priori*, on the basis of postulated pathways of change in the SASA! logic model, to reflect the broad range of community-level impacts expected as a result of the intervention [23]. Within four areas of impact, six outcomes were defined:

#### Reduced social acceptance of gender inequality and IPV

- Acceptability of IPV (among all women; all men)
- Acceptability that a woman can refuse to have sex (among all women; all men)

#### Decrease in experience of IPV

- Past year experience of physical violence from a partner (among women who have had an intimate partner in the past year)
- Past year experience of sexual violence from a partner (among women who have had an intimate partner in the past year)

#### Improved response to women experiencing violence

- Appropriate community response to women experiencing physical and/or sexual IPV in the past year (among women who experienced physical and/or sexual IPV in the past year)

#### Decrease in sexual risk behaviors

- Past year concurrent sexual partners (among non-polygamous partnered men)

Details of questionnaire items used to construct outcomes, and hypothesized directions of the intervention effect on each, are presented in Table 1. Questions on IPV were the same as those used in the World Health Organization (WHO) Multi-country Study on Women’s Health and Domestic Violence [25], and similar to those in the Uganda Demographic and Health Survey [26]. Questions on attitudes were originally taken from the WHO Multi-country study and then adapted and added to in order to increase their validity and reliability within this setting. Items used to measure respondents’ views on the acceptability of a man’s use of violence against his female partner were further revised between baseline and follow-up in order to increase the validity of the indicator, as it was felt that under-reporting of attitudes accepting of violence had occurred at baseline, especially among men. The question on acceptability of a woman refusing sex with her partner was also simplified so as to capture underlying acceptability of sex-refusal rather than its acceptability in specific circumstances such as sickness. Hence comparisons between baseline and follow-up prevalence cannot be made for indicators of attitudes. Appropriate community response to IPV was recorded if a woman with past year experience of physical and or sexual IPV reported that someone in the community tried to help them while the experiences were happening or afterwards, and did so with at least one of a range of appropriate responses reflecting actions encouraged by the intervention (ranging from direct intervention during episodes of violence, to asking the woman how she wants to be helped, to informing a CA or other authority figure about the violence).

**Table 1:** **Questionnaire items used to construct outcomes**

	**Indicator**	**Respondents (denominator)**	**Items in composite indices**	**Expected direction of change due to intervention**
**Social acceptance of gender inequality and IPV**	Acceptability of physical violence by a man against his partner	Men; Women	Answers ‘yes’, a man has good reason to hit his wife in at least one of the following scenarios: • She disobeys him • She answers back to him • She disrespects his relatives • He suspects that she is unfaithful • He finds out she has been unfaithful • She spends time gossiping with neighbours • She neglects taking care of the children • She doesn’t complete her household work to his satisfaction • She refuses to have sex with him • She accuses him of infidelity • She tells his secrets to others in the community • He is angry with her	Decrease
	Acceptability of a woman refusing sex	Men; Women	Answers that ‘yes’ in their opinion it is acceptable if a married woman refuses to have sex with her husband if she doesn’t feel like it.	Increase
**Women’s past year experience of IPV**	Past year experience of physical IPV	Women who have had a regular partners/casual partner in the past year	Reports that her partner/most recent partner has done at least one of the following things to her in the past year: • Slapped her or thrown something at her that could hurt her • Pushed her or shoved her or pulled her hair • Hit her with his fist or something else that could hurt her • Kicked her, dragged her or beat her up • Choked or burnt her on purpose • Threatened to use or actually used a gun, knife or other weapon against her • Threatened to use or actually used a panga (stick) against her	Decrease
	Past year experience of sexual IPV	Women who have had a regular partners/casual partner in the past year	Reports that her partner/most recent partner has done at least one of the following things to her in the past year: • Forced her to have sexual intercourse by physically threatening her, holding her down or hurting her in some way • She had sexual intercourse because she was intimidated by him or afraid he would hurt her	Decrease
**Response to women experiencing violence**	Appropriate community response to women experiencing IPV in past year	Women who report in the survey having experienced physical and/or sexual IPV in the past year	Reports that during or after the experience, ‘yes’ someone in their community tried to help them AND they did so with at least one of the following responses: • Gathered other people from the community to help • Knocked on their door to stop the fighting • Separated her and her partner during the fighting • Informed a community activist, ssenga, LC or police or other authority • Talked to her afterwards and asked her how she wanted them to help her • Told her to talk to someone else such as a family member, friend, community activist, LC, ssenga or other authority figure	Increase
**Sexual risk behaviour**	Past year concurrent sexual partners among men partnered in the past year	Non-polygamous men who report having had a regular partner in the past year	Answers ‘yes’ to having had a sexual relationship with any other women in the last 12 months, while being with his partner/most recent partner.	Decrease

### Study precision

Sample size, at both the cluster and individual level, was decided upon with the aim of conducting the highest powered study deemed feasible given resource, staffing and geographical constraints surrounding intervention implementation and data collection. Precision estimates (in the form of 95% confidence intervals (CIs)) for measures of effect of the most distal primary outcomes (IPV and concurrent partners) were calculated on the basis of projected sample sizes for a range of values of outcome prevalence, effect sizes and inter-cluster variance (coefficient of variation (k)) [27]. Considering these estimates, a baseline target sample size was set at four communities and 800 respondents per arm (100 men and 100 women per site) [see Additional file 3].

At follow-up, the study received increased funding for the survey, and so the target sample size was increased to 1,200 respondents per arm (150 men and 150 women per site). This decision was taken not only to increase study precision, but also to allow for higher powered secondary analyses of sub-groups in order to understand better the differential intervention effects and explore pathways of change.

From the outset, oversampling of households was used to achieve target sample sizes, to allow for households without an eligible member and potential refusals. At baseline, 2,240 households were sampled, with the aim of completing 1,600 interviews (800 men and 800 women, split evenly between intervention and control sites). At follow-up, 3,360 households were sampled, with the aim of completing 2,400 interviews. More detail on sample size is provided in the Study Protocol [23].

We also recognized that the study would yield effect estimates with wide CIs (including unity) if effect sizes were modest or levels of inter-cluster variance high [23]. Nevertheless, we chose the cluster randomized trial (CRT) design over an individually focused evaluation because: (1) community interventions require community-level evaluation; (2) randomization minimizes important sources of bias, such as program placement bias and self-selection bias; and (3) the cluster level analysis adequately takes into account the clustered nature of the data. The value of this study is thus in the provision of unbiased effect estimates for a broad range of outcomes which, assessed alongside postulated pathways of change, allow us to assess the consistency and coherence of results across different indicators, and the plausibility that they are a result of the intervention [28].

### Statistical analysis

Data were double-entered into a purpose built Microsoft Access database, containing range and logic checks, and discrepancies between twin-entries were resolved with reference to source data. Statistical analysis was performed using Stata version 12.

As pre-specified in the study protocol [23], the primary analysis was done at the cluster level, on an Intention to Treat (ITT) basis - data on all respondents were included according to the site they lived in regardless of whether or not they reported any contact with the intervention. The analysis followed the basic principles for the analysis of CRTs as set out by Donner and Klar [29], using a two-staged approach similar to that used in several recent studies evaluating community-based HIV and violence prevention interventions in Africa [12,30–32].

Crude measures of intervention effect (prevalence ratios) were calculated to compare the intervention group with the comparison group at follow-up; site-level prevalence measures were entered into an analysis of variance model that included terms for intervention and site-pair. Statistical weighting, with weights inversely proportional to the variance of each measure, was applied to all site-level summaries to account for differences in denominators between sites. For the community response outcome, one site recorded no instances of the outcome; therefore, 0.5 was added to allow calculation of a log prevalence.

The generation of adjusted prevalence ratios involved two stages. First, an individual logistic regression model, in which the dependent variable was the outcome of interest, was fitted to data from control villages. Independent variables included age, marital status and baseline EA-level prevalence of the outcome measure of interest (or closest proxy measured at baseline) fitted as a continuous predictor. This model was used to predict the number of people in each site who would be expected to experience the outcome at follow-up in the absence of the intervention. For each site, the ratio of observed to expected (O/E) numbers with the outcome was then calculated. These site-level ratios were then entered into an analysis of variance model including terms for intervention and site-pair, and as with the crude analysis, statistical weighting was applied.

Due to the small number of clusters in the study, we also conducted a sensitivity analysis using an unpaired t-test to compare outcomes between intervention and control groups, thereby increasing statistical power while still producing valid results [33].

A secondary analysis was conducted to assess intervention effect among those in SASA! communities who reported at least a threshold level of exposure to the intervention. The assessment of individual exposure was made based on responses to survey questions on the number of times a respondent had seen a given set of SASA! materials and the number of times they had attended different types of activities. Exposed individuals from intervention sites were matched to individuals in control sites using propensity score matching (using the optmatch2 command in Stata; see study protocol for more detail) [23]. Intervention and control sites were then compared using the same cluster level approach as in the primary analysis, but with site-level summaries including only intervention recipients and their matched controls.

Since CRTs of complex community interventions are often restricted to a small number of clusters, and since behavior change linked to established attitudes and norms is difficult to achieve within project timeframes, evaluations such as this often have limited power to obtain statistically significant results. Therefore, as outlined in the study protocol [23], when interpreting results our emphasis will be, not only on the statistical significance of individual results, but on assessing whether observed intervention effects occurred in the hypothesized direction and the magnitude of these effects. In particular, if observed effects across all outcomes are in the expected direction and largely coherent with one another, this will build a plausible case for intervention impact on the intended outcomes [28]. Conversely, statistically non-significant effect estimates, some in the hypothesized direction and others in the opposite direction would provide less convincing evidence.

The study is registered at ClinicalTrials.gov (reference number NCT00790959) and the study protocol peer-reviewed and published in the journal *Trials* [23].

## Results

### Intervention delivery

Monitoring data show that over the course of the study, CEDOVIP staff supported over 400 activists to implement SASA! in their communities. They led more than 11,000 activities, which took a variety of formats, including community conversations, door-to-door discussions, quick chats, trainings, public events, poster discussions, community meetings, film shows and soap opera groups. Using ongoing process and monitoring data, Raising Voices and CEDOVIP estimate that SASA! activities reached more than 260,000 community members (unpublished data).

During the study, there were some unexpected disruptions to SASA! implementation. Around the time of the presidential and parliamentary elections of February 2011, CEDOVIP had to suspend implementation for almost four months as it became difficult to engage community members without being accused of partisanship. A further three month suspension of activity occurred when police banned people from congregating in groups of more than five people following violent clashes between security forces and members of the public during opposition campaigns against the results of the election. While CEDOVIP countered the resulting loss of momentum by intensifying activities and increasing staff presence once programming resumed, these interruptions meant that intervention communities only received an estimated 2.8 years of SASA! programming during the four year study period.

### Response rates and trial profile

**Figure 3:**
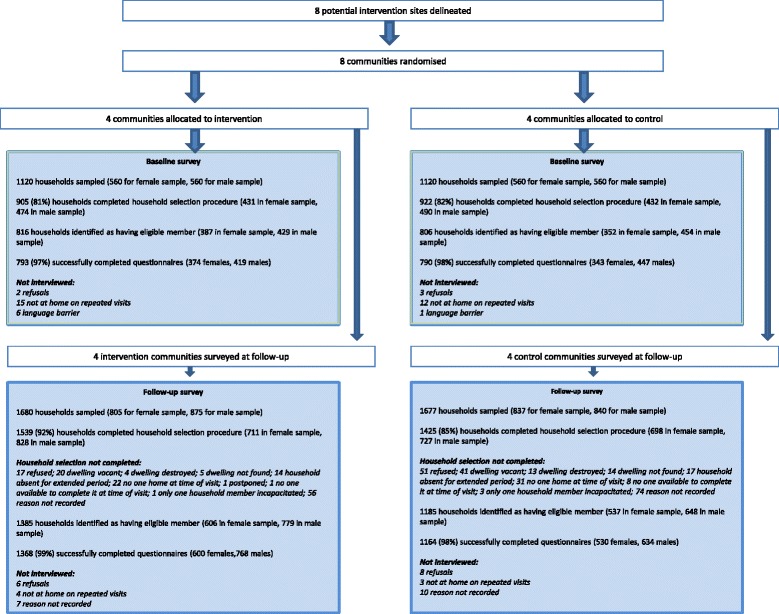
**Trial profile.**

Response rates for both the baseline and follow-up surveys were high (Figure 3). At baseline, 374 women and 419 men were successfully interviewed in intervention communities (97%), and 343 women and 447 men in control communities (98%). At follow-up, 600 women and 768 men were interviewed in intervention communities (99%), and 530 women and 634 men in control communities (98%).

Tables 2 and 3 show the characteristics of study sites and survey respondents at baseline and follow-up, demonstrating high levels of comparability between intervention and control communities at both time-points. At follow-up most respondents lived in rented houses, with the majority of households reliant on basic drinking water and sanitation facilities. A total of 85% of households had access to electricity. Baganda was the most represented tribe, with Catholicism the most prominent religion, followed by Protestantism, Islam and Born Again Christianity. Approximately a third of women and a quarter of men had not progressed beyond primary education. Women were approximately three times more likely than men to report not earning an income, with around a third of women reporting no income. Women were also more likely than men to have ever had a regular partner (more than 90% of women compared to 76% of men) and reported slightly higher levels of marriage or cohabitation (59% versus 51%). Some differences were observed between intervention and control communities. Intervention sites had a larger average population size than control sites (mean at follow-up of 3,190 versus 1,811 households), with considerable growth occurring across all sites over the course of the study. At follow-up, the mean age of respondents in intervention communities was approximately one year greater than in control communities.

**Table 2:** **Site-level characteristics at baseline and follow-up**

**Site-level characteristics**	**Baseline**		**Follow-up**	
	**intervention** **mean** ^**a**^ **(range)**	**Control** **mean** ^**a**^ **(range)**	**intervention** **mean** ^**a**^ **(range)**	**Control** **mean** ^**a**^ **(range)**
Number of sites	4	4	4	4
Number of CAs per site	two sites with 8 (4 female, 4 male)	two sites with 8 (4 female, 4 male)	two sites with 8 (4 female, 4 male);	two sites with 8 (4 female, 4 male);
	two sites with 16 (8 female, 8 male)	two sites with 16 (8 female, 8 male)	two sites with 16 (8 female, 8 male)	two sites with 16 (8 female, 8 male)
Number of households per site (in sampling frame)	1,866 (852 to 2,648)	1,367 (974 to 1,829)	3,190 (1,866 to 4,465)	1,811 (1,444 to 2,526)
% of households with electricity	74 (65 to 80)	79 (67 to 89)	85 (82 to 89)	85 (83 to 86)
% of households where main drinking water source is a public tap	63 (52 to 80)	68 (57 to 80)	65 (42 to 80)	64 (53 to 80)
% of households using traditional pit toilet/latrine	63 (57 to 74)	60 (55 to 64)	57 (49 to 67)	54 (45 to 61)
% of households living in rented accommodation	65 (47 to 79)	71 (59 to 82)	76 (68 to 86)	72 (64 to 82)
% belonging to Baganda Tribe	72 (64 to 77)	65 (35 to 80)	66 (57 to 71)	62 (38 to 78)
% belonging to main religions				
Catholic	36 (29 to 40)	36 (30 to 43)	37 (34 to 38)	36 (31 to 40)
Muslim	25 (21 to 31)	26 (21 to 32)	24 (19 to 29)	22 (16 to 29)
Protestant	23 (17 to 31)	24 (22 to 25)	25 (18 to 29)	26 (25 to 29)
Born Again	13 (10 to 17)	10 (9 to 13)	13 (10 to 16)	13 (10 to 19)

^a^Unweighted mean of site-level summary data.

**Table 3:** **Characteristics of respondents to baseline and follow-up surveys**

	**Baseline**				**Follow-up**			
**Individual-level**	**Men number (%)**		**Women number (%)**		**Men number (%)**		**Women number (%)**	
	**Intervention**	**Control**	**Intervention**	**Control**	**Intervention**	**Control**	**Intervention**	**Control**
Age (years) - mean(sd)	27.1 (6.8)	27.6 (7.0)	28.4 (7.7)	28.2 (7.7)	28.6 (7.8)	29.9 (8.2)	28.4 (7.4)	29.1 (8.2)
Above primary education	275/419 (66%)	321/447 (72%)	157/374 (42%)	140/343 (41%)	556/768 (72%)	457/634 (72%)	394/599 (66%)	343/529 (65%)
Does not earn money	87/419 (21%)	94/447 (21%)	180/374 (48%)	166/343 (48%)	108/768 (14%)	63/634 (10%)	219/599 (37%)	177/529 (33%)
Ever had a regular partner	326/418 (78%)	352/447 (79%)	350/374 (94%)	316/342 (92%)	584/768 (76%)	481/634 (76%)	558/599 (93%)	487/529 (92%)
					Including casual: 689/768 (90%)	Including casual: 573/634 (90%)	Including casual: 574/599 (96%)	Including casual: 497/529 (94%)
Had a regular partner in past 12 months	313/419 (75%)	335/447 (75%)	305/374 (82%)	274/343 (80%)	545/768 (71%)	435/634 (69%)	486/599 (81%)	401/529 (76%)
					Including casual: 624/768 (81%)	Including casual: 525/634 (83%)	Including casual: 504/599 (84%)	Including casual: 427/5292 (81%)
Currently married/cohabiting	165/419 (39%)	191/447 (43%)	228/374 (61%)	205/343 (60%)	407/768 (53%)	314/634 (50%)	377/599 (63%)	286/529 (54%)
In polygamous marriage (among those married)	37/165 (22%)	45/191 (24%)	49/201 (24%)	57/187 (30%)	36/407 (9%)	38/314 (12%)	53/316 (17%)	57/246 (23%)
No children	237/419 (57%)	223/447 (50%)	83/374 (22%)	83/343 (24%)	351/768 (46%)	267/634 (42%)	136/599 (23%)	121/528 (23%)

### Intervention exposure

Very few respondents in control communities reported any exposure to SASA! materials, activities or multi-media events (2% of men and 1% of women), a reassuring indication that efforts to reduce diffusion of the intervention to control communities were successful. In the intervention communities, exposure to SASA! was higher among men than among women. A total of 91% of men compared to 68% of women reported any exposure to materials, activities or multi-media events, with prevalence of exposure varying somewhat between sites (range for men, 89% to 95%; for women, 59% to 88%). A total of 85% (81% to 92%) of men versus 53% (44% to 73%) of women reported exposure to all three routes (materials, activities, multimedia events) at least once, or at least one route once and another route at least a few times.

### Impact on primary outcomes

Table 4 presents data on community-level intervention/control comparisons for the primary outcomes assessed in this trial. Baseline and follow-up data are presented for each outcome, alongside crude and adjusted risk ratios (and 95% CIs) comparing the prevalence of the outcome in the intervention communities versus control communities at follow-up. All differences between intervention and control communities, as indicated by the adjusted risk ratios, were in the hypothesized direction of intervention effect, with large effect sizes and CIs excluding unity for many of the indicators.

**Table 4:** **Estimates of effect on primary outcome indicators** ^**a**^ **, comparing outcome in intervention versus control communities**

**Primary outcome indicators**	**Baseline**		**Follow-up**			
	**Intervention**	**Control**	**Intervention**	**Control**	**Unadjusted RR** ^**a**^ **(95% CI)**	**Adjusted RR** ^**b**^ **(95% CI)**
**Reduced social acceptance of gender inequality and IPV**						
Acceptability of physical violence by a man against his partner						
• Male attitudes	112/419 (27%)	107/445 (24%)	136/768 (18%)^c^	544/634 (86%)^c^	0.13 (0.01 to 1.19)	0.13 (0.01 to 1.15)
• Female attitudes	214/373 (57%)	203/343 (59%)	191/599 (32%)^c^	311/528 (59%)^c^	0.54 (0.37 to 0.79)	0.54 (0.38 to 0.79)
Acceptability that a woman can refuse sex						
• Male attitudes	223/419 (53%)	251/447 (56%)	744/768 (97%)^c^	474/634 (75%)^c^	1.31 (0.98 to 1.77)	1.31 (1.00 to 1.70)
• Female attitudes	152/374 (41%)	123/342 (36%)	542/599 (90%)^c^	385/529 (73%)^c^	1.26 (1.04 to 1.53)	1.28 (1.07 to 1.52)
**Decrease in women’s experience of IPV**						
Past year physical IPV	75/302 (25%)	57/273 (21%)	46/504 (9%)	93/424 (22%)	0.45 (0.14 to 1.46)	0.48 (0.16 to 1.39)
Pasty year sexual IPV	38/303 (13%)	31/273 (11%)	70/504 (14%)	84/423 (20%)	0.76 (0.33 to 1.74)	0.76 (0.33 to 1.72)
**Improved response to women experiencing IPV**						
Appropriate community response to women experiencing IPV in past year	-	-	28/102 (27%)	18/139 (13%)	1.91 (0.46 to 7.94)	2.11 (0.52 to 8.59)^d^
**Decrease in sexual risk behaviors**						
Past year concurrent sexual partners among non-polygamous men partnered in past year	109/270 (40%)	105/284 (37%)	139/508 (27%)	177/397 (45%)	0.60 (0.35 to 1.02)	0.57 (0.36 to 0.91)

^a^Risk ratios calculated at the cluster-level, both crude and adjusted ratios adjusting for community-pair, and weighted according to the number of observations per village. ^b^Adjusted risk ratios generated on the basis of expected number of events from a logistic regression model on individual data with independent variables including age, marital status and EA-level summary baseline measure of outcome indicator.

^c^Attitudinal outcomes were revised between baseline and follow-up to provide more valid measures - a baseline/follow-up comparison is therefore not possible. ^d^Baseline measure controlled for: disclosed past year IPV and got helpful response. CI, confidence interval; IPV, intimate partner violence; RR, risk ratio.

Both women and men in intervention communities were more likely than their control counterparts to have progressive attitudes. In intervention communities, social acceptance of a man’s use of violence against his partner was significantly lower among women (adjusted risk ratio 0.54, 95% CI 0.38 to 0.79) and lower among men (0.13, 0.01 to 1.15). Similarly, more people in intervention communities reported attitudes supporting the acceptability of a woman refusing sex, statistically significant for both women (1.28, 1.07 to 1.52) and men (1.31, 1.00 to 1.70).

Past year experience of physical IPV was substantially lower among intervention women compared to control women (0.48, 0.16 to 1.39). However, there was a much higher level of inter-site variation for this indicator among control communities at follow-up than was seen at baseline, reducing our power to obtain a statistically significant result for this indicator when analyzed at the cluster level (despite the large effect size). For sexual IPV, the difference between intervention and control communities was somewhat smaller and statistically non-significant (0.76, 0.33 to 1.72).

Among women reporting past year experience of physical and/or sexual IPV, the intervention was associated with a more than two-fold greater appropriate community response to this violence (2.11, 0.52 to 8.59). However, due to a small denominator (women experiencing IPV in the past year) and considerable inter-site variation for this outcome, the CI around this estimate is very wide, making it difficult to draw conclusions as to the true intervention effect on this outcome.

Men in intervention communities were considerably less likely to report having had concurrent sexual partners in the past year compared to men in control communities, and this result was statistically significant (0.57, 0.36 to 0.89).

The unpaired T-tests produced point estimates and CIs very similar to the paired analysis, with the exception that intervention effect on male attitudes regarding the acceptability of violence by a man against his partner became statistically significant (0.13, 0.02 to 0.73).

Most measures of effect did not change substantially when the analysis was restricted to men and women reporting at least moderate levels of exposure to SASA! (and their control counterparts matched on propensity for exposure) (see Table 5).

**Table 5:** **Estimates of effect on primary outcome indicators** ^**a**^ **– comparison of results from primary and secondary analyses**

**Primary outcome indicators**	**Primary analysis – including all individuals in communities**	**Secondary analysis – including individuals exposed to SASA!, and matched controls** ^**b**^
	**Adjusted RR** ^**a**^ **(95% CI)**	**Adjusted RR** ^**a**^ **(95% CI)**
**Reduced social acceptance of gender inequality and IPV**		
Acceptability of physical violence by a man against his partner		
• Male attitudes	0.13 (0.01 to 1.15)	0.09 (0.01 to 1.24)
• Female attitudes	0.54 (0.38 to 0.79)	0.44 (0.30 to 0.63)
Acceptability that a woman can refuse sex		
• Male attitudes	1.31 (1.00 to 1.70)	1.32 (1.02 to 1.72)
• Female attitudes	1.28 (1.07 to 1.52)	1.37 (1.14 to 1.65)
**Decrease in women’s experience of IPV**		
Past year physical IPV	0.48 (0.16 to 1.39)	0.57 (0.32 to 1.03)
Past year sexual IPV	0.76 (0.33 to 1.72)	0.78 (0.41 to 1..49)
**Improved response to women experiencing IPV**		
Appropriate community response to women experiencing IPV in past year	2.11 (0.52 to 8.59)^c^	3.53 (0.91 to13.62)^c^
**Decrease in sexual risk behaviors**		
Past year concurrent sexual partners among non-polygamous men partnered in past year	0.57 (0.36 to 0.91)	0.53 (0.32 to 0.87)

^a^Risk ratios calculated at the cluster-level, both crude and adjusted ratios adjusting for community-pair, and weighted according to the number of observations per cluster. Adjusted risk ratios generated on the basis of expected number of events from a logistic regression model on individual data with independent variables including age, marital status and EA-level summary baseline measure of outcome indicator. ^b^Matched on propensity for exposure, with propensity for exposure predicted using a logistic regression model including age, marital status, duration of relationship, duration living in community, whether stayed elsewhere in past year, work and its location, time spent out in community, whether live in gated compound, community pair, % of EA households in gated compounds, number of households in EA. ^c^Baseline measure controlled for: disclosed past year IPV and got helpful response. CI, confidence interval; EA, Enumerated Areas; IPV, intimate partner violence; RR, risk ratio.

### Inter-cluster variation

Levels of inter-cluster variation for each outcome changed somewhat between baseline and follow-up, as indicated by estimates of k (the coefficient of variation of the prevalence between clusters) at each time point. Values for an unmatched study are presented here in place of k_m_ (the coefficient of variation within matched pairs), so as to be able to comment on these changes using data from all clusters at baseline and only control clusters at follow-up (data from intervention sites cannot be used to calculate coefficients of variation at follow-up since the variation between clusters would in part be due to the intervention effect itself). The most marked increases in inter-cluster variation over the course of the study were seen for the IPV outcomes, particularly physical IPV, while marked decreases in variation were observed in relation to male reports of the acceptability of a man’s use of violence against his partner, and past year concurrency. Coefficients of variation were as follows: acceptability of violence, among men (Baseline (BL) k = 0.46, Follow-up (FU) k = 0.045), and among women (BL k = 0.098, FU k = 0.20); acceptability of a woman refusing sex with her partner, among men (BL k = 0.16, FU k = 0.14), and among women (BL k = 0.18, FU k = 0.14); past year physical IPV (BL k = 0, FU k = 0.45); past year sexual IPV (BL k = 0.19, FU k = 0.33); appropriate community response (BL community response not measured, FU k = 0.38); concurrent sexual partners (BL k = 0.21, FU k = 0).

## Discussion

The SASA! Study assessed the community level effect of a community mobilization intervention on the social acceptance of gender inequalities and IPV, prevalence of IPV, community responses to IPV and sexual risk behaviors. Intervention impacts were observed in the hypothesized direction for all primary outcomes assessed. Most strikingly, deeply entrenched behaviors shifted, with women’s past year experiences of physical IPV and men’s past year concurrency approximately 50% lower in intervention communities compared to control communities. The magnitude of effect on sexual IPV was smaller, in accordance with our hypothesis that both attitudes and behaviors regarding sexual IPV would be harder to shift [23], but nevertheless the effect estimate was in the desired direction. Importantly, and in contrast to most current evidence, these intervention effects are demonstrated at the community level, and are not limited to those with high reported levels of intervention exposure. This attests to the success of the community diffusion process at the heart of the intervention model. It is also suggestive of the importance of the multiple strategies and social levels through which the intervention may have its intended impacts (for example, through community responses to violence in addition to personal change within relationships).

The intervention was successfully delivered. High numbers of community members in the quantitative survey reported exposure through varied routes, and monitoring and evaluation data and qualitative data (to be presented elsewhere) indicate high levels of CA activity.

The SASA! Study has several strengths. It is the first CRT in sub-Saharan Africa to assess the community impact of a gender focused structural IPV and HIV prevention intervention. Cluster randomization removes the potential for program placement bias, with community matching ensuring that intervention and control communities are similar despite the small number of sites randomized. Furthermore, we attempted to control for neighborhood bias by standardizing the process of recruiting volunteers (as loci of the community survey sample) across both intervention and control sites. Using an ITT analysis on data from a random sample of community members, we assessed the overall community impact of the intervention rather than effects among self-selecting individuals choosing to participate in intervention activities. The repeated cross-sectional design allowed us to control for potential baseline imbalances in the prevalence of the outcomes between intervention and control communities. Measurement bias was minimized through use of a standardized questionnaire administered by interviewers who had undergone three weeks of intensive training on conducting surveys related to IPV and sexual behavior.

Intervention development and implementation, along with the statistical analysis plan, were informed by a pre-specified conceptual framework of pathways of change and intervention impact. We are thus able to assess the consistency, congruency and coherence of observed changes in primary outcome indicators in relation to this framework [23]. Subsequent papers will explore a range of secondary outcomes relating to broader gender norms, communication within relationships, other types of controlling behaviors and abuse, female empowerment, HIV-related behaviors, and community responses to violence, to further understand the range of intervention impacts and potential pathways of effect.

The study also had several limitations, with a number of factors potentially biasing estimates of intervention effect towards the null. Since social diffusion is at the heart of the SASA! intervention, and the overall study area is small, it is likely that some undetected contamination of control sites occurred, despite the geographical buffers between sites (and despite low levels of reported exposure among control respondents). Furthermore, external interruptions to programming during the course of the study, along with the fact that some of the study communities experienced moderate levels of population mobility, mean that levels of intervention exposure might not have been optimum among survey respondents nor had time to take effect on deeply entrenched behaviors. Results must therefore be interpreted as the short-term community-wide effects of SASA!, rather than measures of the potential efficacy of the intervention given ideal experimental conditions. Despite this we were still able to observe sizeable effects on most of our outcomes.

As with many trials of community-based interventions, the number of communities included was small and the precision of some effect estimates is therefore low. Despite this, 95% CIs excluded 1 for most of the attitudinal outcomes and the sexual concurrency outcome. It is also worth noting that, while levels of physical IPV declined in intervention communities over the course of the study, inter-cluster variation for this outcome increased markedly in control sites. This additional heterogeneity was unexpected and as the statistical power of a CRT is strongly determined by the degree of inter-cluster variation, it weakened the power of the study to detect a statistically significant intervention impact on the IPV outcome.

Reporting bias is a potential limitation in a study of attitudes and behaviors around IPV. While under-reporting of IPV is common, it is possible that increased sensitization to issues surrounding IPV and its disclosure will have disproportionately increased reports of IPV experience among women in intervention communities. Again, this would result in our effect estimate being lower than the true intervention effect. Conversely, among men, increased sensitization to the issues may lead to the under-reporting of negative behaviors and over-reporting of progressive attitudes in intervention communities, thereby leading us to over-estimate intervention effects on male outcomes. However, if social desirability bias has some role in influencing our observed results, this at least indicates a positive shift in perceived social norms in accordance with SASA!’s objectives to achieve community level norm change.

In relation to the sexual IPV outcome, it is important to note that while women in intervention communities reported lower levels of sexual IPV than their control counterparts, reports of sexual IPV did not go down in intervention sites during the course of the study, rather they increased in control communities. The reason for this is not fully understood, although one hypothesis is that external factors increased awareness across both intervention and control sites of what constitutes sexual coercion and, therefore, led to increased overall reporting across study communities. Several factors are worthy of mention in this respect. In Uganda as a whole, violence against women has received more attention in the media in recent years. The Domestic Violence Act, passed in 2010, led to considerable national debate on the subject of marital rape, and may have contributed to an increased recognition of sexual IPV and concomitant improvements in support available and willingness to talk about sexual violence within intimate partnerships. In addition to these exogenous factors, during the course of implementing SASA!, CEDOVIP worked with police and healthcare workers around the provision of post exposure prophylaxis (PEP) (for HIV), to encourage and support health facilities to provide PEP and post rape treatment to people reporting sexual violence. Related materials were highly circulated and present in all police stations, health units and Local Council offices (across both intervention and control communities), potentially also adding to a climate in which sexual IPV was discussed more openly. If this hypothesis is valid, the fact that reports of sexual IPV did not increase in SASA! communities despite general background increases in rates of disclosure, might indicate that SASA! did indeed bring actual levels of violence down. Increased recognition of the concept of sexual coercion within intimate partnerships may also explain why at follow-up a high proportion of respondents in control communities also report progressive views regarding the acceptability of a married woman refusing sex with her husband.

### Lessons for the field

This is the first CRT within sub-Saharan Africa to show that community mobilization can have meaningful community-level impacts within project timeframes, and the study findings have a number of important implications for donors and development partners.

For donors and organizations that work to prevent violence against women and HIV, the study highlights the value of investing in social norm change interventions at the community level by engaging with both men and women at all levels of the community structure. For many organizations, the focus on prevention at the community level represents a departure from their current prevention programming which is commonly focused upon services for those experiencing violence or on prevention through individual- rather than community-level change. Another innovation is the explicit focus on power rather than gender. The decision by Raising Voices to make discussions of power and power inequalities a central focus of the intervention arose out of a recognition that an initial explicit focus on ‘gender’ is likely to be off-putting to many. This shift away from the language of women’s rights and gender is credited with helping to make SASA! more relevant, thought provoking and interesting for community members, as well as broadening the scope of potential intervention impacts beyond those strictly related to violence against women. As all community members are likely to have been disempowered at some point in their lives, this focus supports the broader engagement of both women and men in intervention activities inviting them to consider their own power and be more conscious about how they use it in all kinds of interactions. Ultimately, the use of an entry point of power leads to discussions about gender inequality and violence, but these topics emerge from the analysis of who holds power in the community and how it may be misused, rather than being imposed on the community from the outset.

The study findings also have important implications for the field of HIV prevention. Impacts on sexual concurrency, as well as the social acceptance of and prevalence of violence, both of which are associated with increased HIV risk, illustrate the potential importance for HIV prevention of aspirational messaging about relationships beyond communicating knowledge about the HIV risks of multiple partnerships to improving levels of communication, trust and intimacy within relationships. These issues are at the heart of HIV vulnerability, yet seldom addressed in HIV programming.

More broadly, the study yields important lessons for the field of violence and HIV prevention intervention research. Firstly, it is feasible to conduct CRTs of community mobilization interventions, even where numbers of clusters are restricted. Secondly, this study was only possible because of a strong partnership between the research and implementation partners. This partnership meant that we were able to design the study around a clear understanding of the intervention and its aims, set up and maintain the CRT design in an ethically responsible way, feed research findings back into the program in an ongoing manner, ensure that the control communities were able to receive the intervention following study completion, and develop programmatically relevant conclusions from the research.

## Conclusions

This is the first CRT in sub-Saharan Africa to assess the community level impact of a community-level violence and HIV prevention program. The findings suggest that SASA! achieved important community-level impacts over programmatic timeframes, with the results consistent with positive intervention impacts on all of the primary outcomes assessed. This is an important advancement for the field of violence and HIV research and strongly supports further replication of the intervention. SASA! is now being delivered in the control communities and replicated in more than 15 countries. Future analysis will explore the costs of the intervention, the broader impacts of SASA!, and the processes of change occurring within communities and couples.

## Additional files

Additional file 1:
**SASA! An Activist Kit for Preventing Violence Against Women and HIV.** Description: Detailed description of the SASA! intervention. Additional file 2:
**Intervention logic model.** Description: Detailed description of intervention logic model. Additional file 3:
**Precision estimates for effect sizes given varied assumptions about control-arm prevalence and between community variation.** Description: Table of precision estimates for effect sizes used to inform decisions about study sample size.

## Abbreviations

aRR: adjusted risk ratio
CA: community activist (in the SASA! Intervention)
CEDOVIP: Centre for Domestic Violence Prevention
CI: confidence interval
CRT: cluster randomized trial
DHS: Demographic and health survey
EA: Enumeration Area
IPV: intimate partner violence
ITT: intention to treat analysis
