# Supplementary material for: Findings from the SASA! Study: a cluster randomized controlled trial to assess the impact of a community mobilization intervention to prevent violence against women and reduce HIV risk in Kampala, Uganda
Source: BMC Med. 2014 Jul 31;12:122. doi: 10.1186/s12916-014-0122-5 (PMC4243194; doi:10.1186/s12916-014-0122-5)
Supplement: Additional file 1: — SASA! An Activist Kit for Preventing Violence Against Women and HIV. Description: Detailed description of the SASA! intervention. [file 12916_2014_122_MOESM1_ESM.docx]

## Additional file 1: SASA! An Activist Kit for Preventing Violence Against Women and HIV

Raising Voices and the Centre for Domestic Violence Prevention (CEDOVIP) are NGOs in Uganda that have been working in the field of violence prevention for over a decade. SASA! was designed by Raising Voices, and was implemented in Kampala by CEDOVIP. SASA! is a community mobilisation intervention that seeks to change community attitudes, norms and behaviours that result in gender inequality, violence and an increased HIV vulnerability for women. Designed around the Ecological Model of violence, [[32](#_ENREF_32), [33](#_ENREF_33)] SASA! recognises that intimate partner violence results from the complex interplay of factors which operate at the individual, relationship, community, and societal levels, and that if effective change is to be achieved, it is important for interventions to systematically work with a broad range of stakeholders within the community. In the SASA! intervention CEDOVIP staff work with four groups of actors: community activists (CAs) selected from the more progressive men and women rooted in the community, who work voluntarily to facilitate and promote SASA! activities; community leaders including Ssengas (traditional marriage counselors) who, as religious, cultural, governmental and other types of local leaders, are encouraged to integrate a gender and power analysis into their leadership roles; professionals such as health care providers and police officers, who provide direct prevention and response services; and institutional leaders who have the power to implement policy changes within their institutions.^[[1]](#footnote-1)^ SASA! entails the selection, training, and ongoing mentoring and skill building of these individuals and groups, to help improve their knowledge and inspire their activism to engage their social networks and different spheres of influence to address gender inequality and violence.

Recognising that an initial, explicit focus on ‘gender’ is likely to be off putting to many, the central focus of the intervention is to promote a critical analysis and discussion of power and power inequalities. As all community members are likely to have been disempowered at some point in their lives, this focus supports the broader engagement of both women and men in intervention activities. The intervention aims to be aspirational, and support a critical analysis not only of the ways in which men and women may misuse power, and how this affects their intimate relationships and the community, but also on how people can use their power positively to affect and sustain change at an individual and community level. Ultimately the use of an entry point of power leads to discussions about gender inequality and violence, but these topics emerge from the analysis of who holds power in the community and how it may be misused, rather than being imposed on the community from the outset.

Using this operational model, SASA! aims to support a phased, community level process of change, analogous to the processes set out in the individual-level behavior Stages of Change Theory of Prochaska et al. (1992)[[34](#_ENREF_34)], with the four phases (Start, Awareness, Support, Action). Each phase builds on the other, with an increasing number of individuals and groups involved in each phase strengthening a critical mass committed to and able to create social norm change. During the first phase (Start), CEDOVIP staff focus on strengthening the capacity and ‘power within’ of CAs and other key stakeholders to work on issues of violence and gender - engaging them in critical thinking and discussion about what constitutes violence; the causes and consequences of violence; the underlying links between violence, gender inequality and the misuse of power; and the implications for individuals, families and communities. Gender inequality and social norms about sexual behavior for men and women are also discussed and opened up to analysis. Time is also spent getting to know more about the community’s perceptions of violence against women, gender and HIV, and building relationships with leaders and key gate-keepers who will support and enable the community mobilization in the subsequent phases.

During the second phase (Awareness) and subsequently, CAs and leaders are supported by CEDOVIP staff to conduct a range of local activism activities, including door-to-door visits, interactive community dramas, film shows, poster discussions, public events and one-on-one ‘quick chats’. The process of engagement for this and subsequent phases is done in an informal manner, with CAs integrating the activities into their day-to-day lives in their communities with neighbors, friends, and other groups to which they belong. The intention of this awareness phase is to spark and actively diffuse critical thinking among community members about men’s use of ‘power over’ women and the community’s silence about this. The aim is for a range of different community members to engage in discussions about power, and the ways in which power imbalances between men and women help perpetuate violence against women and HIV/AIDS risk, and question the legitimacy of violence against women and gender inequality. Alongside this, local leaders, the police, health workers and other professionals receive training and support to improve their community-based prevention efforts and the provision of services. At an institutional level, leadership of police and health care sectors are engaged in a series of seminars introducing similar ideas and analysis of the role their sector could play in addressing violence against women.

In the third phase (Support), community members are encouraged to explore alternatives to the status quo that would create more gender equality, power balance and happiness in their families and communities. The concept of joining ‘power with’ others is explored through the local activism activities - power to create positive change, for community members to reach out and support women experiencing violence and couples trying to balance power, and to challenge men using violence. Activities focus upon helping people to develop appropriate skills to reduce inequities in their relationships, and to challenge and respond appropriately to violence in their communities. These activities seek to encourage recognition of the ways in which different individuals can address the misuse of power, gender inequality and violence, and the strength that can be generated when they join together with a common aim. Community leaders and professionals are supported to work more closely together to address violence and gain skills in preventing and responding to violence against women. At an institutional level, policies and practices are examined by leaders with the support of CEDOVIP staff to identify areas where changes could be made to increase the capacity of the police and health sector to meaningfully respond to violence against women.

The focus of the final phase (Action) aims to consolidate and normalize a greater sharing of power and non-violence, demonstrating the benefits of more equal relationships, and as a result, preventing violence against women and reducing HIV/AIDS risk. The thrust of this phase is to encourage community members, leaders, professionals and institutions to use their ‘power to’ take action to address gender inequality and violence. Special emphasis is placed on formalizing change within community groups, local leadership structures, service delivery points and institutions.

1. Due to the trial, the media engagement and national advocacy piece was not done so as to limit contamination of control communities. [↑](#footnote-ref-1)
