# Supplementary material for: Findings from the SASA! Study: a cluster randomized controlled trial to assess the impact of a community mobilization intervention to prevent violence against women and reduce HIV risk in Kampala, Uganda
Source: BMC Med. 2014 Jul 31;12:122. doi: 10.1186/s12916-014-0122-5 (PMC4243194; doi:10.1186/s12916-014-0122-5)
Supplement: Additional file 2: — Intervention logic model. Description: Detailed description of intervention logic model. [file 12916_2014_122_MOESM2_ESM.docx]

**Additional file 2: Intervention logic model**

The intervention logic model maps out the key contextual variables that may influence intervention impact; the levels of SASA! activities conducted in different spheres of influence; the expected initial, intermediate and longer term outcomes of the intervention; and the long-term sustained impact the intervention is designed to have on the community.

Contextual variables include both socio-demographic variables and factors such as levels of problematic alcohol use – factors that may be associated with both the occurrence of IPV- and HIV-related risk behaviours, but are not in themselves a focus of the SASA! intervention. For example, place of residence and levels of mobility may influence the degree to which community members are exposed to SASA! – in communities where many people live in gated communities, for instance, it might be more difficult for SASA! to achieve substantial coverage.

The Logic model then details the range of people and community structures involved in and reached by SASA! activities – including individual women and men in the community, people in potential positions of influence who may have a role in helping to prevent or respond to violence (such as neighbours, elders and relatives), as well as people in positions of authority with the potential to provide support to women or men who turn to them for help. The logic model also details the hypothesized initial outcomes, intermediate outcomes and impacts.
