# Supplementary material for: Findings from the SASA! Study: a cluster randomized controlled trial to assess the impact of a community mobilization intervention to prevent violence against women and reduce HIV risk in Kampala, Uganda
Source: BMC Med. 2014 Jul 31;12:122. doi: 10.1186/s12916-014-0122-5 (PMC4243194; doi:10.1186/s12916-014-0122-5)
Supplement: Additional file 3: — Precision estimates for effect sizes given varied assumptions about control-arm prevalence and between community variation. Description: Table of precision estimates for effect sizes used to inform decisions about study sample size. [file 12916_2014_122_MOESM3_ESM.docx]

**Additional file 3: Precision estimates for effect sizes given varied assumptions about control-arm prevalence and between community variation**

| **Outcome** | **Estimated final sample size per site (with 4 intervention and 4 control sites in total)** | **Estimated prevalence of endpoint measure** | | **Effect estimate** | **Estimate of precision of percentage risk difference (95% CI)*** | | | |
| --- | --- | --- | --- | --- | --- | --- | --- | --- |
|  |  | Control arm (%) | Intervention arm (%) | Risk difference (%) | K=0.1 | K=0.2 | K=0.3 | K=0.4 |
| Past year experience of physical IPV (women partnered in past year) | 75 [based on 100 respondents per site, 80% having partner in past year and 5% non-response to violence questions] | 30 | 22.5 | 7.5 | *-2.4 – 17.4* | *-5.2 – 20.2* | *-8.8 – 23.8* | *-12.9 – 27.9* |
|  |  | 30 | 15 | 15 | **5.8 – 24.2** | **3.4 – 26.6** | **0.2 – 29.8** | *-3.4 – 33.4* |
|  |  | 20 | 15 | 5 | *-3.2 – 13.2* | *-4.7 – 14.7* | *-6.9 – 16.9* | *-9.4 – 19.4* |
|  |  | 20 | 10 | 10 | **2.4 – 17.6** | **1.1 – 18.9** | *-0.8 – 20.8* | *-3.0 – 23.0* |
| Past year experience of sexual IPV (women partnered in past year) | 75 [based on 100 respondents per site, 80% having partner in past year and 5% non-response to violence questions] | 15 | 11 | 4 | *-3.1 – 11.1* | *-4.1 – 12.1* | *-5.6 – 13.6* | *-7.3 – 15.3* |
|  |  | 15 | 7.5 | 7.5 | **0.9 – 14.1** | **0.0 – 15.0** | *-1.3 – 16.3* | *-2.8 – 17.8* |
|  |  | 10 | 7.5 | 2.5 | *-3.4 – 8.4* | *-3.9 – 8.9* | *-4.8 – 9.8* | *-5.8 – 10.8* |
|  |  | 10 | 5 | 5 | -0.4 – 10.4 | -0.9 – 10.9 | -1.7 – 11.7 | -2.6 – 12.6 |
| Concurrent sexual partners (among non-polygamous men partnered in the past year) | 75 [based on 100 respondents per site, 80% having partner in past year and 5% non-response to sexual behaviour questions] | 50 | 37.5 | 12.5 | **0.0 – 25.0** | *-5.7 – 30.7* | *-12.5 – 37.5* | *-19.7 – 44.7* |
|  |  | 50 | 25 | 25 | **13.4 – 36.6** | **8.4 – 41.6** | **2.4 – 47.6** | *-3.9 – 53.9* |
|  |  | 40 | 30 | 10 | *-1.3 – 21.3* | *-5.5 – 25.5* | *-10.7 – 30.7* | *-16.3 – 36.3* |
|  |  | 40 | 20 | 20 | **9.5 – 30.5** | **5.9 – 34.1** | **1.3 – 38.7** | *-3.7 – 43.7* |

*Based on calculations provided by Hayes and Bennet (1999)[[28](#_ENREF_28)]

NB: Figures in italics represent situations where the study would not find a statistically significant difference between intervention and control groups.
